# Giant retroperitoneal liposarcoma with colonic infiltration as a cause of gastrointestinal bleeding: Case report and literature review

**DOI:** 10.1016/j.ijscr.2025.111142

**Published:** 2025-03-12

**Authors:** Sebastian Forero-Escobedo, Sandra Milena Gonzalez-Rodriguez, Jose Alejandro Ramírez-Rincón, Vanessa Alejandra Garcia-Bernal, Santiago Polanco-Perdomo, Sofía Echeverri-Torrents

**Affiliations:** a Faculty of Medicine, National University of Colombia, Bogotá, Colombia; b Faculty of Medicine, Pontifical Xavierian University, Bogotá, Colombia

**Keywords:** Soft tissue sarcoma, Dedifferentiated tumor, Colonic invasion, Retroperitoneal neoplasia, Gastrointestinal hemorrhage, Oncological surgery

## Abstract

**Introduction:**

Retroperitoneal liposarcomas are rare malignant tumors that often present asymptomatically until advanced stages. Giant liposarcomas exceeding 30 cm are particularly uncommon, and colonic infiltration causing gastrointestinal bleeding is extremely rare, with only a few cases documented in the literature.

**Case presentation:**

A 78-year-old female presented with hematochezia, constipation, and diffuse abdominal pain. Imaging revealed a large left para-aortic retroperitoneal mass compressing the colon. Colonoscopy showed colonic mucosal atrophy without active bleeding. Surgical exploration via midline laparotomy identified a bilobulated 89 × 35 × 40 cm retroperitoneal mass, infiltrating the left colon and compromising adjacent structures. En bloc resection, including hemicolectomy and left salpingo-oophorectomy, was performed. Histopathology confirmed a dedifferentiated liposarcoma with well-differentiated areas and colonic invasion reaching the muscularis propria. Margins were positive (R1). The patient recovered well postoperatively and experienced a single episode of intestinal obstruction at 6 months, resolved medically.

**Discussion:**

Retroperitoneal liposarcomas typically exhibit compressive behavior rather than invasion. However, dedifferentiation is associated with aggressive features, including local invasion and higher recurrence rates. Colonic infiltration by giant liposarcomas is exceedingly rare, with only three additional cases reported. Mechanisms of bleeding include mucosal ischemia, compression, or direct histological infiltration, as observed in this case.

**Conclusion:**

The relationship between gastrointestinal bleeding and colonic infiltration in retroperitoneal liposarcomas may reflect the association between tumor dedifferentiation and invasive behavior. This underscores the importance of curative-intent surgical management as the primary therapeutic strategy, despite the challenge posed by the proximity to critical neurovascular structures.

## Highlights

• Giant retroperitoneal liposarcomas exceeding 30 cm are exceedingly rare.
• Tumor dedifferentiation links retroperitoneal liposarcomas to aggressive invasive behavior.
• Gastrointestinal bleeding is a rare clinical manifestation of retroperitoneal liposarcomas.
• Complete surgical resection (R0) is essential but challenging due to tumor size and proximity to critical structures.

## 1 Introduction

Retroperitoneal liposarcomas (RL) are rare malignant neoplasms, accounting for approximately 0.07 % to 0.2 % of all malignancies and about 20 % of soft tissue sarcomas [1,2]. Giant retroperitoneal liposarcomas (GEL), defined as those with a diameter exceeding 30 cm or a weight greater than 20 kg, are exceptionally uncommon [3]. These masses are often detected incidentally or at advanced stages due to their anatomical location and slow growth [4]. Despite their size, histological invasion of adjacent organs is infrequent. The estimated incidence is 4 % [5,6], as they generally exhibit a compressive rather than infiltrative behavior [7].

Gastrointestinal bleeding (GB) as a clinical manifestation of RL is extremely rare, and the combination of colonic invasion (CI) and bleeding is particularly unusual. This scenario highlights the previously described association between dedifferentiated histological components and worse clinical outcomes, including histological invasion, local recurrence, and distant metastases [3]. Following the SCARE 2023 guidelines, we present the case of a 78-year-old patient with a giant retroperitoneal liposarcoma manifesting as lower GB and histologically invading the left colon [8].

## 2 Case presentation

**Fig. 1:**
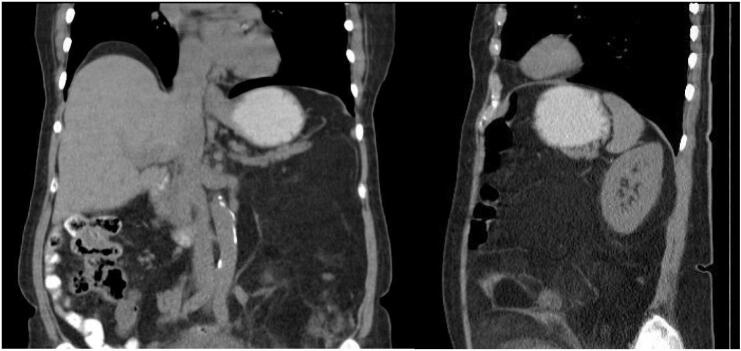
CT in coronal and sagittal views: a left para-aortic retroperitoneal mass is identified, compressing the descending colon against the anterior wall. In both views, the mass extends from the subphrenic region to the pelvis.

**Fig. 2:**
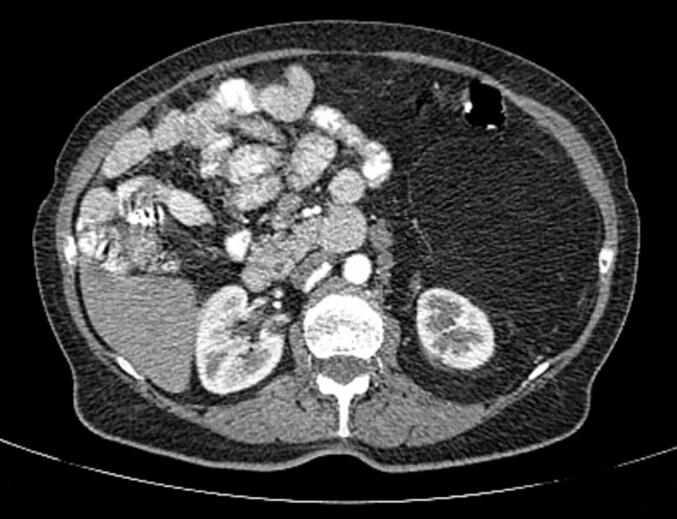
CT with both intravenous and oral contrast. Mass displacing the intestinal loops to the right and excluding the left colon anteriorly, with close relation to the Gerota's fascia and proximity to the left kidney.

A 78-year-old female patient presented with a history of constipation associated with occasional hematochezia and diffuse abdominal pain; and ultrasound revealed a large mass in the left flank with a lipomatous appearance, prompting referral to the oncological surgery service. On physical examination, the patient exhibited a distended, tender abdomen with a palpable mass that did not seem to involve the abdominal wall. Abdominal Computed Tomography (CT) reported a left para-aortic retroperitoneal mass (Fig. 1A), extending from the subphrenic region to the pelvis, compressing the left colon against the anterior wall (Fig. 1B) and displacing the intestinal loops to the right without involving them (Fig. 2). The mass was in close relation to the Gerota fascia and the kidney, with no apparent vascular involvement. A chest CT was performed, which was negative for metastatic lesions.

The patient was electively scheduled for a hemicolectomy and retroperitoneal resection. However, while awaiting the procedure on an outpatient basis, who reconsults in the emergency department with a self-limiting presentation of abundant episodes of hematochezia and bright red rectal bleeding, with stable hemoglobin levels (12.3 g/dL). Colonoscopy findings showing: small patchy areas in the descending colon with smooth, atrophic mucosa, no active bleeding, and what appears to be a bulging caused by extrinsic compression.

**Fig. 3:**
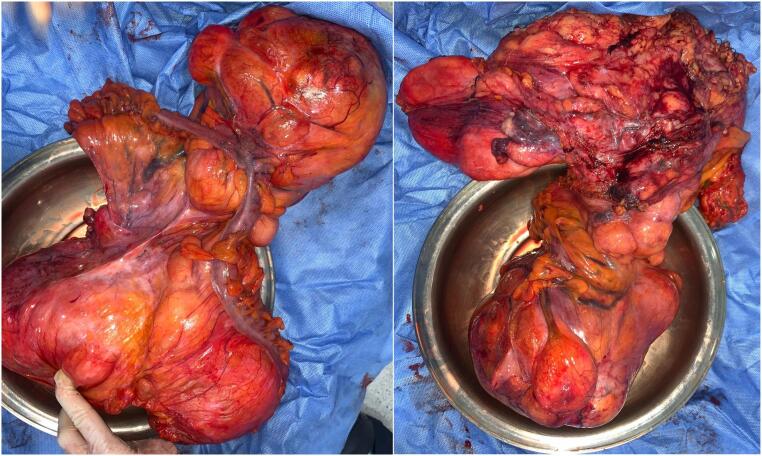
Surgical resection specimen. Anterior views of a bilobed mass that deforms and is strongly adhered to the colon, with areas appearing to infiltrate it. The retroperitoneal section margin shows a heterogeneous surface with necrotic-appearing areas.

Surgically, the patient underwent a midline xiphopubic laparotomy, which revealed a bilobed retroperitoneal mass measuring 89 × 35 × 40 cm. The tumor distorted the anatomical pathway, causing bulging of the left colonic wall (Fig. 3) and involving the inferior mesenteric vessels, Gerota's fat, and adnexal structures. A block resection was performed with paravertebral dissection. The aorta, iliac vessels, and left ureter, although in close proximity to the mass, were successfully preserved. The procedure included a hemicolectomy with colorectal anastomosis and a left salpingo-oophorectomy.

**Fig. 4:**
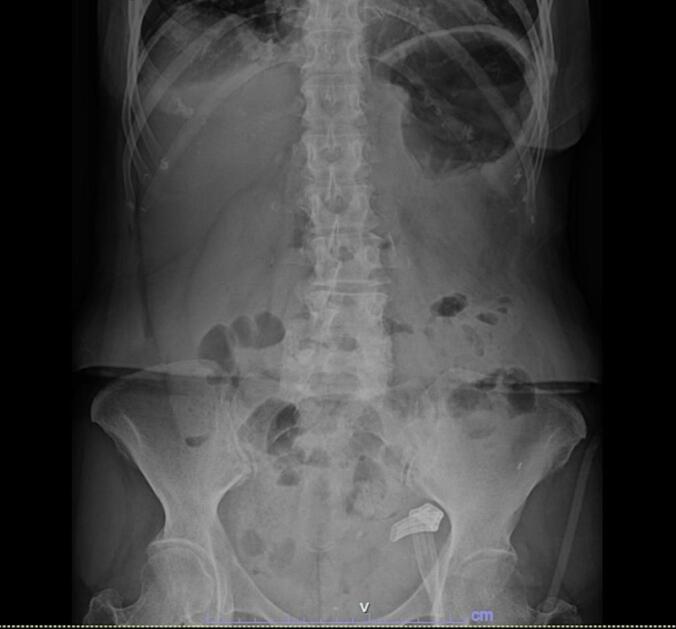
Postoperative radiograph. There is a reduction in intra-abdominal congestion and changes consistent with the colorectal anastomosis.

Postoperatively, the patient recovered without complications (Fig. 4) and was discharged on the eleventh postoperative day. At a six-month follow-up, the patient reported an episode of abdominal pain managed in the emergency department for suspected intestinal obstruction secondary to adhesions, which resolved with medical management.

Histopathology revealed a dedifferentiated liposarcoma with both low- and high-grade components, classified as T4N0M0G3, and assigned a grade 3 according to the FNCLCC (Fédération Nationale des Centres de Lutte Contre le Cancer) classification, with a score of 6/8. Microscopically, the majority of the mass consists of well-differentiated liposarcoma; however, in a macroscopically heterogeneous and necrotic-appearing region, a dedifferentiated component is identified, accounting for approximately 20 % of the overall tumor volume. Up to 19 mitoses were observed per 10 high-power fields (40×), No lymphovascular invasion was evident, and the resected lymph nodes were negative for tumor involvement. Upon sectioning the colon, smooth, atrophic-appearing mucosal areas are observed. Samples of the mucosa adjacent to the mass were taken, revealing microscopically that the tumor extends to the muscularis layer of the colon. The hilum of the left ovary is also found to be invaded by the liposarcoma. A raw area at the retroperitoneal para-aortic resection margin is positive for tumor involvement (R1), measuring 34 × 16 cm.

## 3 Literature review

A literature search was conducted in PubMed and Scopus up to October 12, 2024. The search strategy used was: (“Liposarcoma”) AND (“Retroperitoneal” OR “retroperitoneal space” OR “retroperitoneum”) AND (“colon” OR “colonic” OR “colonic involvement” OR “colonic invasion” OR “gastrointestinal hemorrhage” OR “gastrointestinal bleeding” OR “GI bleeding” OR “Digestive hemorrhage”), and results were filtered for case reports. The inclusion criteria were: a) Primary RL; b) Cases where colonic involvement was observed, whether through compression, adhesion, or infiltration; and c) Presence of GB as a clinical manifestation. Reports with incomplete or unreadable information regarding symptoms or histopathology were excluded.

**Fig. 5:**
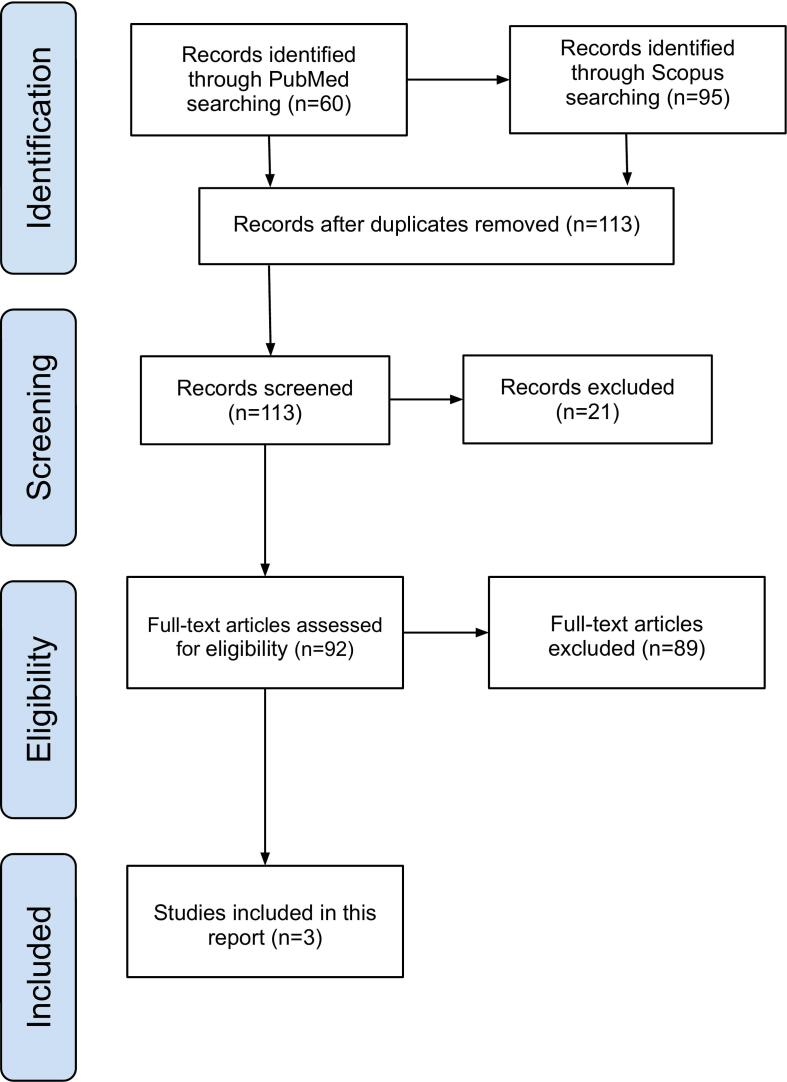
Flow chart of the literature search, identification and selection of the studies.

Fig. 5 shows the flow diagram of article selection. A total of three articles meeting the specified criteria were collected, reported by Wanchick et al. in 2009 [9], Sato et al. in 2014 [5], and Goh et al. in 2021 [10] (Table 1).

**Table 1:** Patients' clinical characteristics.

No.	Author/year	Age/sex	Symptoms/clinical presentation	Preoperative examination	Location/extent of liposarcoma	Source of bleeding	Tumor size	Histology	Treatment
1	Wanchick et al./2009 [9]	70/M	-Rectal bleeding -Nausea, weight loss, abdominal pain and distension	CT, colonoscopy	Compressive effect on the sigmoid colon, encasing terminal ileum, cecum. Involves both kidneys, ureters, bladder.	Significant adhesion to the cecum with invasion of the terminal ileum, (without colonic infiltration).	30 cm × 18 cm × 15 cm	Well-differentiated liposarcoma with areas of dedifferentiation.	SM (R1) resection, adjuvant chemotherapy.
2	Sato et al./2014 [5]	72/M	-Melena	CT, colonoscopy with biopsy	Displaces intestinal loops to the left, seemingly encasing and involving both the kidney and right colon.	Multilobulated lesion protruding into the lumen of the left colon (lesion covered with mucosa).	25 cm × 20 cm × 10 cm	Well-differentiated liposarcoma with a focus of dedifferentiation (infiltration up to the colonic submucosa).	SM (R1) resection
3	Goh et al./2021 [10]	76/F	-Rectal bleeding -Abdominal pain, diarrhea	CT, colonoscopy with biopsy	Mass near the left colon, extending through the inguinal canal and involving the left adnexa.	Ischemic colitis secondary to extrinsic compression of the mass on the colon and its vasculature.	14 cm x X 7 cm × 7 cm	Well-differentiated liposarcoma.	SM
4	Our study	78/F	-Hematochezia, rectal bleeding -Abdominal pain, constipation	CT, colonoscopy	Distorts the left colon pathway, displaces intestinal loops to the right, adheres to Gerota's fascia and left adnexa.	Areas of colonic mucosal atrophy associated with bulging secondary to extrinsic compression.	89 cm × 35 cm × 40 cm	Well-differentiated liposarcoma with a focus of dedifferentiation (infiltration up to the muscularis propria of the colon).	SM (R1) resection

M: male, F: female, CT: Computed tomography, SM: Surgical management, R1: resection with positive microscopic margins.

## 4 Discussion

RL have a late clinical presentation with nonspecific symptoms [3,4]. The difficulty in their initial diagnosis is due to the absence of early symptoms and their indolent growth [7,11]. As a result, most patients with these tumors are in advanced stages at the time of diagnosis, limiting therapeutic options and complicating surgical management [2,12].

Liposarcomas are categorized into well-differentiated, myxoid, dedifferentiated, pleomorphic, and undifferentiated subtypes [2,7]. Dedifferentiated tumors contain well-differentiated lipomatous areas with abrupt transitions to pleomorphic, non-lipogenic sarcomatous components and are associated with a poorer prognosis due to their higher propensity for local recurrence and metastasis [13,14]. This highlights the importance of strict postoperative monitoring, given the high recurrence rate characteristic of dedifferentiated liposarcomas, which may reach as high as 66 % [12].

As a histological classification system, the FNCLCC has been widely used to assess the aggressiveness of soft tissue sarcomas. This system stratifies tumors into three grades (grade 1, grade 2, and grade 3) based on differentiation, mitotic index, and tumor necrosis [15]. The analysis of microscopic findings has proven to be superior to other classifications in predicting survival and metastasis-free or local recurrence-free survival [16,17]. A multicenter study conducted by Bonvalot et al. [18] in 382 patients in France found that, in the analysis of a subset of 290 patients without microscopic residual disease, tumor grade was the only prognostic factor for overall survival. Meanwhile, factors associated with better local control included low tumor grade, compartmental surgery, and the experience of the treating center.

Another study conducted by Gronchi et al. [19] in a retrospective series of 343 patients concluded that grade 1 and 2 sarcomas undergoing early intervention exhibit a more pronounced local benefit, whereas grade 3 tumors are significantly associated with an increased likelihood of developing distant metastases [20].

These neoplasms are likely the tumors with the potential to become the largest in the human body; however, those exceeding 30 cm remain extremely rare [3]. A review conducted by Sun et al. in 2024 [3] indicates that, to date, only 35 cases of GRL have been reported in the English literature. Most of these tumors reached exceptional sizes due to the ample retroperitoneal space, which allows for growth before causing symptoms or compression of neighboring organs [7].

The treatment for RL is based on complete surgical resection, which is the main prognostic factor for recurrence and survival [12]. However, achieving negative margins (R0) can be challenging due to the proximity of critical neurovascular structures such as the aorta, vena cava, and spine, as well as the difficulty in distinguishing normal fatty tissue from well-differentiated neoplastic tissue [11]. In a 13-year retrospective study by Neuhaus et al. [6], involving a cohort of 72 patients, it was reported that in order to achieve macroscopic complete resection with negative microscopic margins (R0) during primary surgery, more than half of the patients required combined resection of surrounding organs, with the colon being resected in 25 % of cases, despite direct histological involvement being rare.

In cases of surgical resections with positive margins, adjuvant therapy has been proposed as a management approach; however, its role in the retroperitoneum remains undetermined [21]. A meta-analysis by Li et al. [22], which analyzed more than 30,000 patients and compared surgical management with and without adjuvant therapy (radiotherapy or chemotherapy), concluded that patients who received adjuvant radiotherapy had a 20 % reduction in the risk of death compared to those who underwent surgery alone. On the other hand, chemotherapy did not improve survival and, in fact, showed a trend—although not statistically significant—toward an 11 % higher risk of death. In patients with localized and resectable recurrences, repeat resection remains the therapeutic approach with the greatest impact on survival, with a median of up to 60 months [23,24]. However, in cases of multiple recurrences, each subsequent laparotomy is associated with increased morbidity and a shorter disease-free interval, making surgical indication a case-by-case decision, particularly in rapidly progressing sarcomas where the expected benefit is limited [23].

Histological infiltration of the colon by primary RL is extremely rare. To our knowledge, including the present study, only 4 cases have been described in the English literature, reported by Sato et al. in 2014 [5], Kim et al. in 2010 [25], and Park et al. in 2020 [26]. It is noteworthy that CI is considerably more common in primary tumors originating in locations close to the colon, such as the mesentery or areas near the serosa, and less frequent in those of retroperitoneal origin due to their expansive nature [27,28].

In this case, the infiltration extended to the muscularis propria of the colon and left ovarian hilum, reinforcing the association between tumor dedifferentiation and invasive behavior [14,29]. Furthermore, the 4 reported cases indicate that CI is often related to large tumors or those located in specific regions of the retroperitoneum that facilitate close contact with the colon, such as the perinephric fat, which serves as a favored site for infiltration [22,29]. The presence of histological multiorgan involvement by proximity (colon and ovarian hilum), along with a grade 3 classification, are likely unfavorable prognostic factors for recurrence, especially in the context of non-achieved R0 surgical margins.

GB is an uncommon manifestation of RL. In the literature review, only three additional cases of GB were identified in patients with RL that had some degree of association or involvement of the colon. Various mechanisms were identified that could explain this clinical presentation. The most evident mechanism is histological infiltration of the colon reaching the mucosa, as described in the case by Sato et al. [5]. However, other mechanisms have also been reported, such as ischemic colitis secondary to vascular compression (Goh et al. [10]), infiltration of another portion of the digestive tract associated with strong adherence to the colon (Wanchick et al. [9]), or prolonged extrinsic compression of the colon leading to partial infiltration of the histological layers and mucosal atrophy, as seen in this case.

## 5 Conclusion

GRL with colonic infiltration is an extremely rare condition that can present, in very specific situations, with lower GB through a variety of both mechanical and histological pathophysiological mechanisms. This case highlights the complexity of these neoplasms, whose diagnosis is often made at advanced stages due to their slow growth and anatomical location. The association between tumor dedifferentiation and invasive behavior observed in this case underscores the importance of a comprehensive surgical approach as the main therapeutic strategy. Although achieving negative margins (R0) can be challenging due to the proximity of critical neurovascular structures such as the aorta, the vena cava, or the spine, complete resection is key to improving prognosis and reducing the high rates of local recurrence.

## Consent

Written consent was obtained from the patient for publication of this report. Any details identifying the individuals to the clinical history and images associated were eliminated as to remain anonymous.

## Ethical approval

Ethical approval is not needed for case reports in our institution.

## Funding

This research did not receive any specific grant from funding agencies in the public, commercial, or not-for-profit sectors.

## Author contribution

Forero Escobedo S conceptualized the study, contributed to data analysis and interpretation, drafted the manuscript, and approved the final version. Gonzalez Rodriguez SM contributed to data collection, data analysis, and manuscript drafting. Ramírez Rincón JA participated in the development of the methodology, data collection, and critical revision of the manuscript for intellectual content. Polanco Perdomo S and Echeverri Torrents S collected data and assisted in data interpretation.

## Guarantor

Forero Escobedo Sebastian

## Research registration number

Not applicable. This case report did not involve a first-time procedure or intervention; therefore, registration is not deemed necessary.

## Declaration of competing interest

The authors declare they have no conflicts of interest.
